# Microhotplate Temperature Sensor Calibration and BIST

**DOI:** 10.6028/jres.116.025

**Published:** 2011-12-11

**Authors:** M. Afridi, C. Montgomery, E. Cooper-Balis, S. Semancik, K. G. Kreider, J. Geist

**Affiliations:** National Institute of Standards and Technology, Gaithersburg, MD 20899-0001; Department of Electrical and Computer Engineering, University of Maryland, College Park, MD 20740; National Institute of Standards and Technology, Gaithersburg, MD 20899-0001

**Keywords:** BIST, calibration, microhotplate, platinum-rhodium, sensor, silicon, substrate, temperature, thermocouple

## Abstract

In this paper we describe a novel long-term microhotplate temperature sensor calibration technique suitable for Built-In Self Test (BIST). The microhotplate thermal resistance (thermal efficiency) and the thermal voltage from an integrated platinum-rhodium thermocouple were calibrated against a freshly calibrated four-wire polysilicon microhotplate-heater temperature sensor (heater) that is not stable over long periods of time when exposed to higher temperatures. To stress the microhotplate, its temperature was raised to around 400 °C and held there for days. The heater was then recalibrated as a temperature sensor, and microhotplate temperature measurements were made based on the fresh calibration of the heater, the first calibration of the heater, the microhotplate thermal resistance, and the thermocouple voltage. This procedure was repeated 10 times over a period of 80 days. The results show that the heater calibration drifted substantially during the period of the test while the microhotplate thermal resistance and the thermocouple-voltage remained stable to within about plus or minus 1 °C over the same period. Therefore, the combination of a microhotplate heater-temperature sensor and either the microhotplate thermal resistance or an integrated thin film platinum-rhodium thermocouple can be used to provide a stable, calibrated, microhotplate-temperature sensor, and the combination of the three sensor is suitable for implementing BIST functionality. Alternatively, if a stable microhotplate-heater temperature sensor is available, such as a properly annealed platinum heater-temperature sensor, then the thermal resistance of the microhotplate and the electrical resistance of the platinum heater will be sufficient to implement BIST. It is also shown that aluminum- and polysilicon-based temperature sensors, which are not stable enough for measuring high microhotplate temperatures (>220 °C) without impractically frequent recalibration, can be used to measure the silicon substrate temperature if never exposed to temperatures above about 220 °C.

## 1. Introduction

Microhotplate-based conductance-type gas sensors have been under development for approximately two decades. Monolithic array implementation, low-power consumption, and low thermal time constants (around 1 ms) make these devices suitable for low-cost, high-performance, gas-sensor applications [1, 2]. Specifically, the short thermal time constant of the microhotplate was exploited to identify different gas species from the response signature of a single microhotplate gas sensor during a series of rapid temperature steps [3, 4]. This technique provides tunable selectivity from a single microhotplate to complement other dimensions of selectivity available from the pattern of response obtained over an array of microhotplates having different gas sensor film compositions. But the potential of this technique and more recent approaches [5] can only be realized if the same temperature profile is used every time. This demands excellent long-term (over a year) stability from the microhotplate temperature sensor. Barrettino et al. [6] identified the importance of the microhotplate temperature sensor and replaced the commonly used polysilicon temperature sensor with a platinum temperature sensor because the calibration of polysilicon temperature sensors drifts over time. However, these authors did not verify the long-term stability of the calibration. Also while this appears to offer a good solution, it does not address BIST functionality, which requires at least two stable temperature sensors, at least one of which must measure absolute temperature rather than temperature difference. Resistance type temperature sensors satisfy this requirement. Furthermore, it is our experience (unpublished) that aluminum, which like polysilicon is available in a standard CMOS process, is also unsuitable as a temperature sensor at temperatures above 300 °C.

For system integration and mass production of microhotplate devices, BIST functionality is required to ensure reliable long-term operation. BIST typically validates critical system specifications during manufacturing and verifies system performance during the normal use of the system. In the case of microhotplate-based gas-sensor systems, the precision and long-term repeatability of the microhotplate temperature measurements are critical system specifications.

The BIST strategy envisioned in this paper requires two long-term stable microhotplate-temperature sensors based on different thermoelectric mechanisms. In this case, microhotplate temperature BIST consists of comparing the temperatures reported by the two different sensors. As long as the absolute value of the difference remains below an application-specific threshold, the average of the two temperatures is considered reliable. But if the absolute value of the difference exceeds the threshold, the system reports an error. The feasibility of this strategy is demonstrated with a novel two-step, long-term calibration procedure. This paper provides more detail and results than a letter summarizing this work that was recently published [7].

## 2. Microhotplate Device Structure

**Fig. 1:**
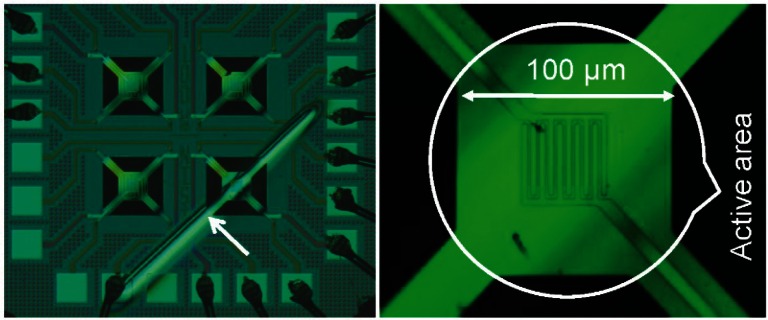
Photographs of the microhotplate test device containing four microhotplates (left). The sputtered rhodium trace used as one leg of a thermocouple is visible over the bottom right microhotplate. The exposed interdigitated platinum traces over the active area of the microhotplate, which were intended as gas sensing film electrodes, and which were used as the other thermocouple leg, are shown magnified on the right. The sputtered rhodium trace is barely visible in this magnified view.

Figure 1 shows a microhotplate test chip containing four microhotplates on the left and an enlarged view of one of these structures on the right side of the figure. This type of microhotplate, which has been described previously [1], is a trampoline-type structure that has four supporting legs to suspend the microhotplate over an etch pit in the silicon substrate. This etch pit thermally isolates the microhotplate. The 100 μm × 100 μm active area of the microhotplate has a Kelvin type polysilicon serpentine heater (underneath and not visible in Fig. 1) with two current leads and two voltage leads. The four-contact arrangement makes it possible to use the polysilicon as a heating element through the current leads and also to utilize the portion of it that occupies the active area of the microhotplate as a temperature sensor by measuring the current passing through the heater leads and voltage across the voltage leads.

To calibrate the polysilicon temperature sensor, a constant 100 μA dc bias current, which is sufficiently low to cause negligible Joule heating, was applied through the current leads, and the voltage across the voltage leads was measured to give the resistance of the active area of the microhotplate heater excluding the legs. The chip was then heated to accurately known temperatures [8], and the heater resistance as a function of temperature was calculated.

The top surface of the microhotplate has exposed platinum interdigitated electrodes as shown in Fig. 1. Originally designed for metal-oxide sensing film conductance measurements, we used them to build a platinum-rhodium thermocouple junction in the active area by sputtering a 200 nm rhodium film line along opposite microhotplate legs using a shadow mask as shown on the left-hand side of Fig. 1. In the expanded view on the right, the rhodium film line is barely visible due to the depth of focus of the microscope at that magnification. Another isolated set of sensing film electrodes may be needed if this type of device is to function as a gas sensor, but it may also be possible to integrate the gas-sensing film electrodes with the thermocouple in a single structure.

The modified microhotplate used in the work reported here has three independent temperature sensors, one based on the electrical resistance of the polysilicon heater, another based on the thermal resistance of the microhotplate legs, and a third based on the thermal emf (electromotive force) of the platinum-rhodium thermocouple. However, neither the thermocouple nor the microhotplate thermal resistance can be directly calibrated as a temperature sensor by heating the entire chip containing the microhotplate because, unlike the heater resistance which responds to absolute temperature, the thermocouple and the thermal resistance respond to the temperature difference between the microhotplate and the substrate on which it is located.

The purpose of this paper is to report the results of an investigation of the long-term stability of calibrations of these three different types of temperature sensors for potential use in precise and accurate microhotplate temperature measurements and in microhotplate-temperature sensor BIST. The paper also describes tests of the suitability of aluminum- and polysilicon-based Resistance Temperature Detectors (RTDs) to measure the temperature of the silicon-substrate, which is required to convert a temperature difference determined with an integrated thermocouple or from the thermal resistance of the microhotplate into the absolute temperature of the microhotplate.

## 3. Microhotplate Temperature Calibration Setup

**Fig. 2:**
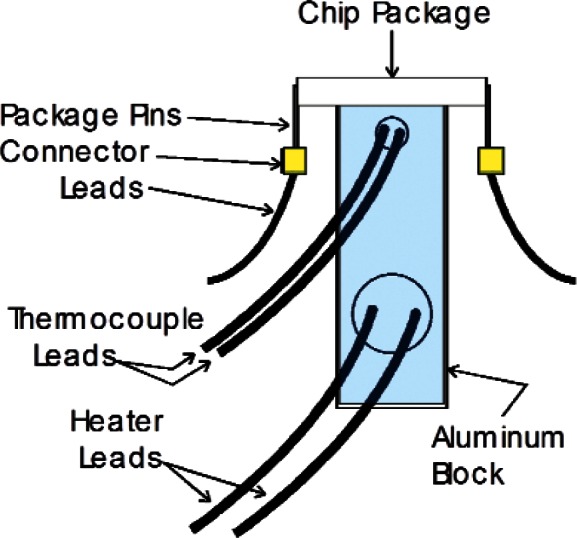
Microhotplate direct temperature-sensor calibration test fixture. The aluminum block shown is suspended in the air by high thermal resistance screws on each side.

Figure 2 shows an end view of a packaged chip mounted on a custom-built temperature-controlled test fixture consisting of an aluminum block containing a heater and a calibrated thermocouple. Screws (not shown) on each end of the aluminum block hold the chip in tight physical contact with the block, which is coated with heat-sink compound. Gold flying-lead connectors are attached to the gold package pins on each side of the block. The heater and the thermocouple also make good thermal contact with the aluminum block through a heat-sink compound that was placed inside the heater and thermocouple wells.

The fixture is interfaced to a characterization system that has connections for the test-fixture heater and thermocouple leads, as well as the flying-lead connections to the package pins. The temperature of the fixture can be raised in programmable step intervals. When the block temperature reaches the desired temperature, which is feedback stabilized with a PID (proportional-integral-derivative) controller, the output voltages from the microhotplate heater are recorded both with and without temperature-sensor bias current. The voltage measured with no bias current is the thermal emf error. The difference between these voltages is the voltage drop across the temperature-sensor resistor due to the bias current.

An upper bound for the difference between the temperature of the aluminum block and the microhotplate, which was located on a die in a ceramic IC package, was measured by attaching a calibrated surface thermometer to the top of a package and comparing its readings with the test-fixture thermocouple readings. The differences were less than 2 °C up to 220 °C. A National Instrument^1^ programming environment was used to develop a virtual-instrument graphical user interface (GUI) to provide automatic data acquisition and control for the characterization system. A Keithley 2400 was used as a programmable constant current source to measure resistance, and a JC Systems Model 600A programmable temperature controller was used to set and hold the temperature of the fixture constant during measurements.

## 4. Long-term Calibration Stability Study

To study the long term stability of the three different microhotplate temperature sensors, 11 experiments were performed over a period of about 3 months in the test bed described above. Each experiment consisted of three steps.

### 4.1 Thermal Stress Treatment

Step 1 of each experiment consisted of holding the temperature of the test-fixture described at 30 °C while applying sufficient power (18 mW) to the microhotplate heater to hold it in the vicinity of 400 °C for a period that varied from 3 days to 16 days. The first thermal stress period was used to anneal the freshly fabricated microhotplate structure. The remaining stress periods simulated the thermal stress that would occur in the normal operation of a microhotplate-based gas sensor in a typical application.

### 4.2 Heater Temperature-Sensor Calibration

**Fig. 3:**
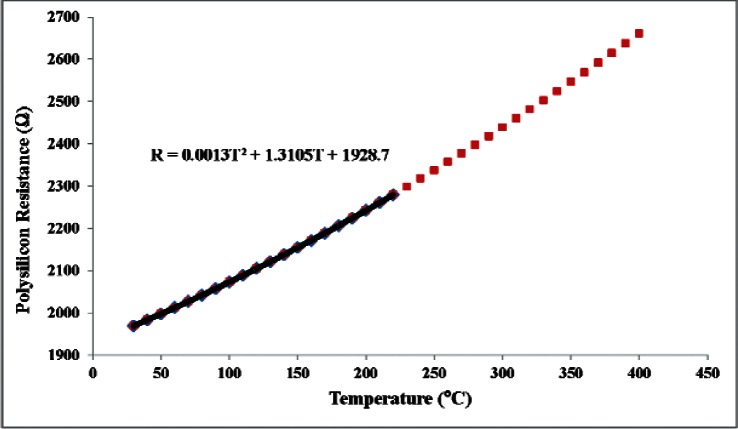
The first resistance verses temperature calibration curve of the polysilicon heater located on the active area of the microhotplate. A second degree polynomial was fit to these data to extrapolate the data to 400 °C for later use in the microhotplate thermal resistance and thermocouple calibrations.

In Step 2 of each experiment, the microhotplate heater was calibrated as a temperature sensor by heating the entire chip containing the die on which the microhotplate was located in the temperature-controlled fixture described in Sec. 3. A 100 μA constant dc current, which raised the microhotplate temperature above the substrate by less than 0.8 °C at 220 °C, was used to measure the heater resistance. The uncertainty associated with these temperature measurements was ± 2 °C. The calibrations were carried out at the 39 test-fixture temperatures

$$T_{j}=30,40,\ldots ,210,220,210,\ldots ,40,30,{}^{\circ}C,$$

and second-order polynomials

(1) $$R_{n}(T_{j})=A_{n}T_{j}^{2}+B_{n}T_{j}+C_{n}$$

were fit to the microhotplate-heater resistances measured in 11 experiments (n = 1,…, 11) as a function of the measured fixture temperature by adjusting the values of *A_n_*, *B_n_*, and *C_n_* with a least squares fitting utility. The data obtained from the first experiment and the equation that was fit to those data are plotted in Fig. 3. For the purposes of this report, it would have been more straightforward to fit the temperature data directly to the microhotplate-heater resistance data, but even a fifth-order polynomial in the heater resistance did not fit the temperature data as well as the second-order polynomial in temperature fit the heater resistance data. In either case, the polynomial was going to be used to extrapolate data covering 30 °C to 220 °C up to approximately 400 °C, and extrapolations based on the second order polynomial of Eq. (1) appeared substantially more robust than those based on fifth-order polynomial functions of R.

### 4.3 Thermocouple and Thermal Resistance Calibration

In Step 3 of the n^th^ experiment, the resistance of the microhotplate’s heater was measured over the range from 30 °C to approximately 400 °C by passing a current through the heater while maintaining the fixture temperature at 30 °C. The temperature of the micro-hotplate as a function of the measured heater resistance was calculated from the solution to Eq. (1) for T as

(2) $$T_{n}(R_{n})=\frac{-B_{n}\pm \sqrt{B_{n}^{2}-4A_{n}[C_{n}-R_{n}]}}{2A_{n}}.$$

**Fig. 4:**
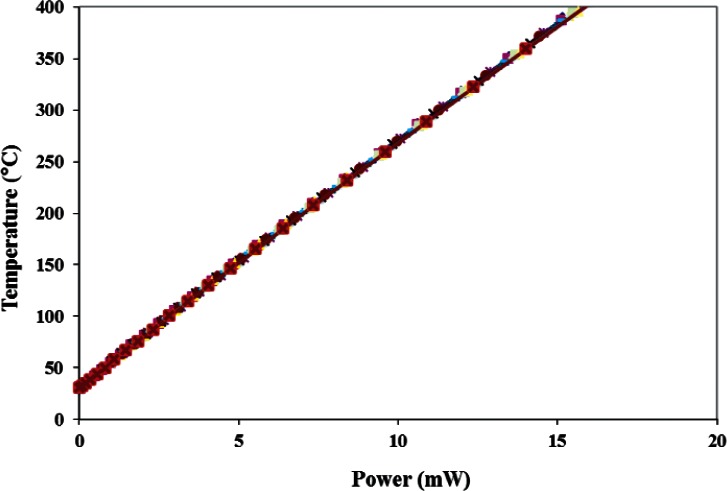
Eleven measurement results of the microhotplate thermal efficiency taken over 80 days show that it remains approximately constant even after a long-period of microhotplate use at elevated temperatures.

**Fig. 5:**
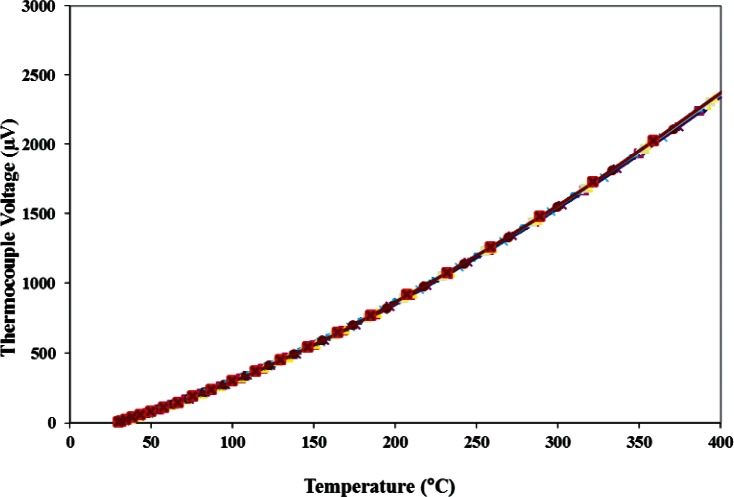
Eleven measurement results of the thermocouple voltage as a function of microhotplate temperature taken over 80 days shows that it remain approximately constant over this period.

Also during Step 3 of each cycle, the power P_n_ being delivered to the microhotplate heater was calculated from the measured microhotplate voltage and current, and the voltage across the platinum/rhodium thermocouple V_n_ was measured as a function of the microhot-plate temperature T_n_ as shown in Figs. 4 and 5, respectively. About 20 min were required for Step 3, with the majority of time spent waiting for the fixture temperature to stabilize after changing the temperature of the microhotplate. This seemed to decrease the variability of the temperature measurements somewhat, particularly at the higher temperatures. In an actual application, corrections for the die temperature would be calculated from simultaneously recorded die-temperature measurements of the type described later in this report, which would eliminate the requirement for a stabilization period.

In Step 3 of the first cycle (n = 1), which occurred on May 9, and only in this cycle, the T_1_ values calculated from Eq. (2) were fit both to the measured heater power P_1_ and to the measured thermocouple voltage V_1_ values. A quadratic equation,

(3) $$T_{P}(P)=DP^{2}+EP+F$$

was sufficient to fit the first experiment temperature versus power data which was almost linear. On the other hand, a fifth order equation,

(4) $$T_{V}=GV^{5}+HV^{4}+IV^{3}+JV^{2}+KV+L$$

was required to fit the first experiment temperature versus thermocouple voltage data, which was quite non-linear.

As mentioned previously, it was not possible to calibrate the thermocouple voltage and microhotplate power as a function of microhotplate temperature using the fixture at different temperatures because these calibrations require a temperature difference between the microhotplate and the die on which it is located. The two-step calibration (microhotplate-polysilicon-heater electrical resistance as a function of fixture temperature followed immediately by microhotplate heater power and thermocouple voltage as a function of polysilicon resistance during heating of the microhotplate with the polysilicon heater) solves this problem without requiring long-term stability of the resistance-versus-temperature calibration of the microhotplate heater.

## 5. Calibration Stability Results

**Fig. 6:**
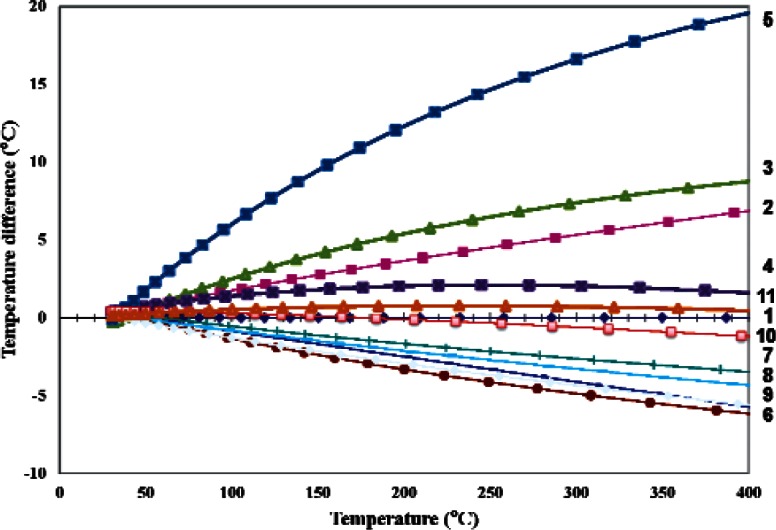
Errors in microhotplate temperature measurements based on a long-term calibration of the microhotplate heater of Fig. 1 as a resistance thermometer at 11 different times over a period of 80 days. The numbers 1 through 11 on the right-side of the graph indicate the experiment numbers that correspond to the data for each experiment.

Assume that the values of A_n_, B_n_, and C_n_ in Eq. (2) do not change during Steps 2 and 3 of the n^th^ experiment. This is a reasonable approximation because the microhotplate remained at a temperature above 200 °C for only about 20 min between the end of Step 2 and the end of Step 3 in any given experiment, compared to the 3 to 16 days during which it was held around 400 °C during Step 1 of the following experiment. With this assumption, the temperature difference T_V_(V_n_) – T_P_(P_n_), which is plotted in Fig. 6 for all 11 experiments, is a measure of the agreement between the temperature measurements based on the thermocouple voltage and the temperature measurement based on the thermal resistance of the microhotplate legs, both of which were based on the original May 9 polysilicon heater resistance calibrations.

As indicated in the figure, the drift shown in Fig. 6 was not a monotonic function of time during the 80 day thermal-stress period. Instead, the temperature-difference curves shown in that figure drifted up and down somewhat erratically during the stability test. It is clear that the lack of temperature-measurement reproducibility evident in Fig. 6 could seriously degrade the ability to distinguish between different gas species and to quantify the concentrations of known gas species in temperature-programmed gas-sensing applications as described in [9].

**Fig. 7:**
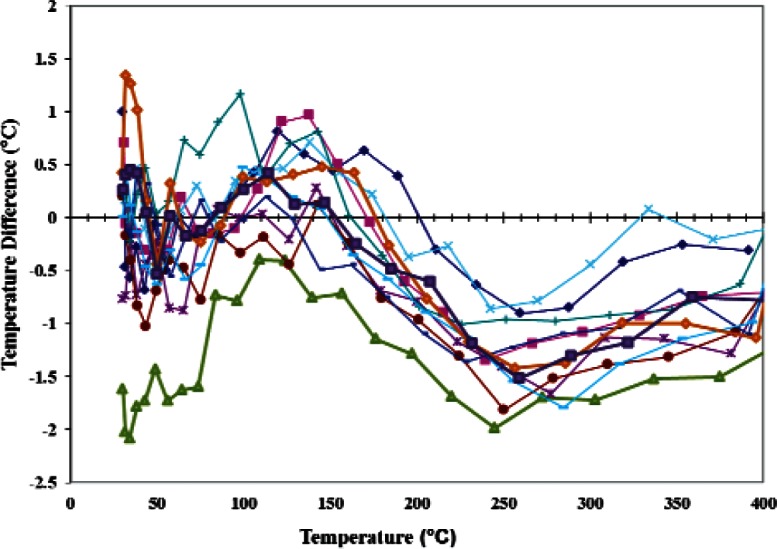
Differences between the microhotplate temperatures based on the long-term calibration of the platinum-rhodium thermocouple and those based on the long-term calibration of the thermal resistance of the heater legs at 11 different times during a period of 80 days.

On the other hand, the temperature difference T_V_(V_n_) – T_P_(P_n_) from Eqs. (3) and (4), which is plotted as a function of the temperature T_n_(R_n_) for the same 11 experiments in Fig. 7, shows that the microhotplate thermal resistance and the thermocouple voltage predict very similar temperatures for the microhotplate during the entire 80 days of measurements. Above 220 °C the absolute uncertainty in the temperature on the abscissa in Fig. 7 is unknown because it is based on an extrapolation. On the other hand, this temperature can be interpreted as an effective temperature, which is precisely reproduced by both the microhotplate thermocouple and thermal resistance of any micro-hotplate. In most applications, a reproducible effective temperature rather than the true temperature is all that is required.

Figure 7 also illustrates the way that temperature sensor BIST would be used in a real application. If repeatability of ± 2 °C were required for some given application, almost all of the temperature measurements on which Fig. 7 was based would be accepted. On the other hand, if ± 1 °C were required, most of the measurements below 220 °C would be accepted and most of those above this temperature would be rejected. However, most of the data that would be rejected fall within a band of ± 1 °C, which suggests that either T_V_(V) or T_P_(P) in Eqs. (3) and (4) does not fit the measured data as well as it could with one more properly chosen adjustable parameters. Therefore, more care in the selection of the fitting functions should enable temperature-sensor BIST at the ± 1 °C for the microhotplate used in this work.

## 6. Substrate Temperature Sensor

The importance of die (microhotplate substrate) temperature measurements was described in Sec. 5. A CMOS test-chip was designed and fabricated with various temperature sensors based on aluminum and polysilicon materials to measure the CMOS silicon substrate temperature. The purpose of this chip design was to test different types of substrate-temperature sensors and to measure their long-term temperature stability in the range of temperatures from ambient to 220 °C. Based on the results of these tests, the most appropriate design (minimum area and/or best long-term stability) substrate-temperature sensor will be chosen for monolithic integration with the microhotplate structures to facilitate temperature-sensor BIST functionality. As pointed out above, the substrate temperature is required to calculate the microhotplate’s active-area temperature from the thermocouple voltage or thermal resistance as they both respond to the temperature difference between the microhotplate and the substrate on which the microhotplate structure is located rather than directly to the microhotplate temperature.

Two different types of substrate temperature sensors were designed, fabricated, and tested for their performance. These were aluminum- and polysilicon-based resistance temperature detectors (RTDs). These substrate-temperature sensors were calibrated using the direct calibration method described in Sec. 3.

Five calibration tests were performed on each test structure in order to assure stability and repeatability of the measurements. These experiments were performed over a period of 6 days. Even though the stress period was not long, the sensors were subject to high temperatures well above their normal operating temperatures, which are unlikely to exceed 80 °C in normal use. The following section describes the design and shows the performance results obtained for the aluminum and polysilicon RTDs.

### 6.1 Aluminum RTD

**Fig. 8:**
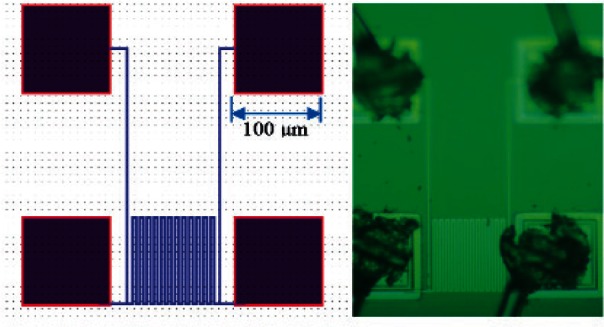
Aluminum-based RTD design for silicon substrate temperature measurements. The design layout (left) and a micrograph of the structure as fabricated (right) are shown.

The aluminum RTD is a four-wire serpentine structure. The design layout and its micrograph are shown in Fig. 8. The four wires are connected to standard 100 μm × 100 μm bonding pads as shown in Fig. 8. To measure the resistance of the sensor as a function of its temperature, two of its wires were used for sourcing a small constant current (100 μA) while the other two were used for measuring the voltages. The device was calibrated and tested for its long-term performance.

**Fig. 9:**
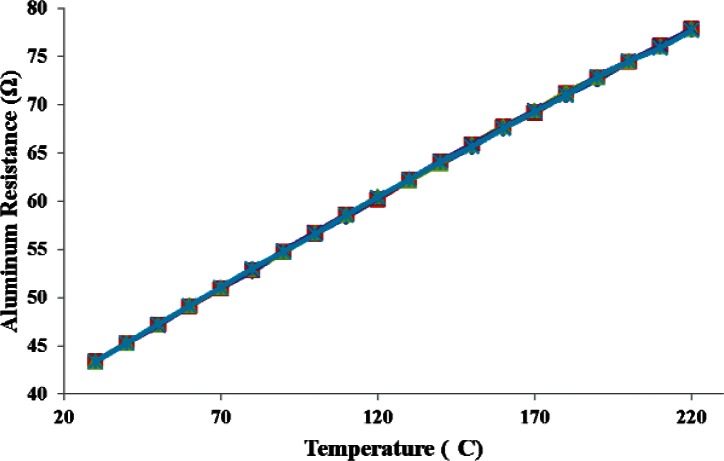
Five substrate temperature calibration curves (shown on top of each other) show the stability and repeatability of the aluminum-based RTD.

Figure 9 plots the five calibration curves for this device. A linear equation fits the data well. The temperature sensor resistance changed from 43 Ω to 78 Ω when the temperature was varied from 30 °C to 220 °C.

**Fig. 10:**
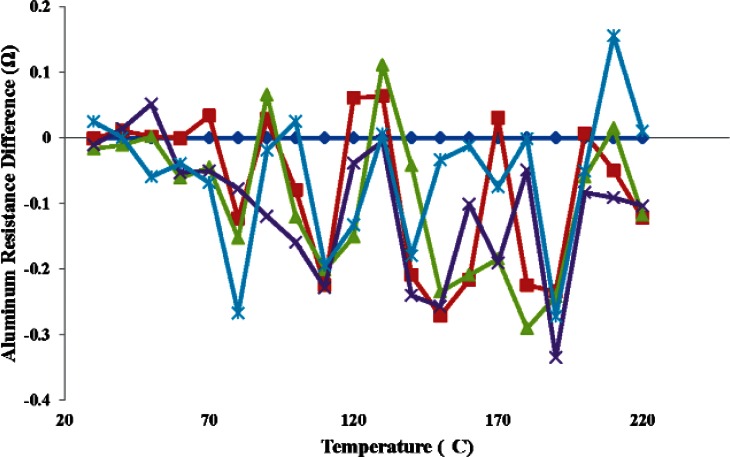
Measured differences among the five substrate temperature calibration curves with respect to the first calibration of the aluminum RDT.

In Fig. 10 the difference of each calibration run is plotted with respect to the first calibration run. The maximum absolute difference from the first measurement encountered was 0.33 Ω at 190 °C, which corresponds to an error of 1.8 °C in temperature. Although the stability data reveal a low error for this device, its size is too large to provide an optimal solution for a substrate temperature sensor.

### 6.2 Polysilicon RTD

**Fig. 11:**
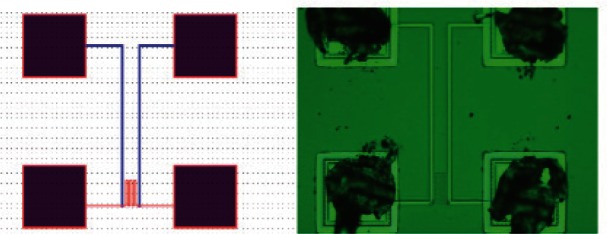
Polysilicon-based RTD design for silicon substrate temperature measurements. The design layout (left) and a micrograph of the structure as fabricated (right) are shown.

The polysilicon RTD is also a serpentine four-wire structure. The four wires are connected to standard 100 μm × 100 μm bonding pads as shown in Fig. 11. To measure the resistance of the polysilicon temperature sensor, two of its pads were used for sourcing a small (100 μA) constant current through the serpentine structure while the other two were used to measure the voltage across it.

**Fig. 12:**
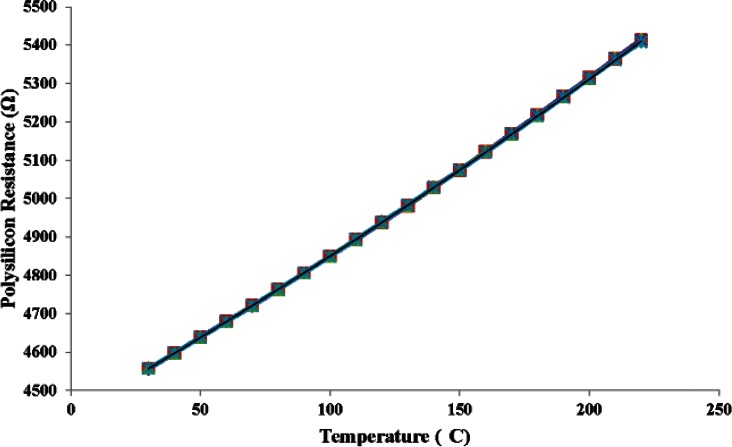
Five substrate temperature calibration curves (shown on top of each other) show sensor stability and repeatability of the polysilicon-based RTD.

**Fig. 13:**
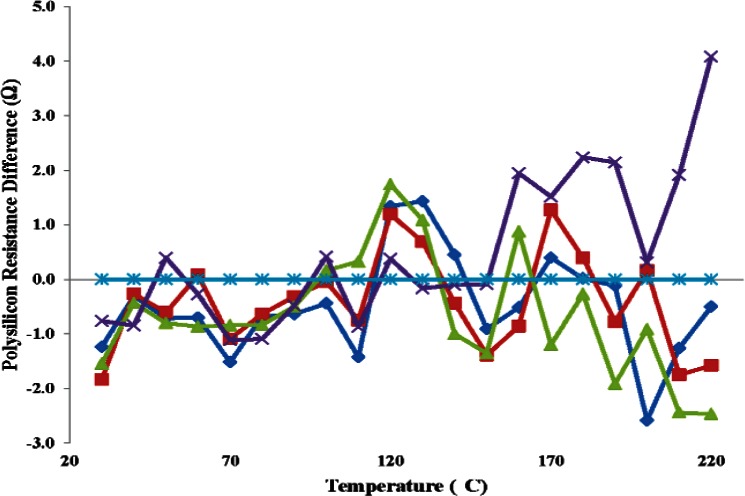
Measured differences among the five temperature calibration curves with respect to the first calibration of the polysilicon RDT.

Figure 12 plots the five calibration curves for this device. A second order polynomial fit these data well. The sensor's resistance changed from 4556 Ω to 5407 Ω when the temperature was varied from 30 °C to 220 °C. Figure 13 plots the difference between each measurement and the first measurement among the group of five measurements. The maximum difference of 4 Ω was recorded at 220 °C. This corresponds to an error of 0.8 °C in temperature. Similarly, the maximum error at 30 °C was about 1.84 Ω which corresponds to an error of about 0.46 °C. The footprint for the polysilicon RTD is about 1/4 th of the aluminum RTD design which makes it a better candidate for integration.

Although we have shown that polysilicon and aluminum are not well suited as materials for temperature sensor implementation in microhotplate structures due to drift in their material properties when they are subject to higher temperatures (>200 °C) for prolonged periods of time, they performed well for measuring the silicon substrate temperature in the temperature range below about 100 °C.

## 7. Conclusions

In this paper we have demonstrated a method to calibrate microhotplate temperature sensors that respond to temperature differences rather than absolute temperatures relative to an absolute microhotplate temperature sensor that is not stable over long periods of time. We have also demonstrated for the first time that microhotplate thermal resistance can be used as a microhotplate temperature sensor, at least for one microhotplate implementation, and have demonstrated the first integration of a thin-film platinum/rhodium thermocouple in a microhotplate structure with stable temperature measurement results.

Based on the results reported here, we tentatively conclude that either an integrated thermocouple or the thermal resistance of the microhotplate can be used with an integrated platinum resistance temperature sensor of the type described in [6] as the basis for microhotplate-temperature BIST. Also based on these results, we further conclude that an integrated thermocouple in combination with the thermal efficiency of the microhotplate can be used with a polysilicon-heater temperature sensor as the basis for microhotplate-temperature BIST even though the later cannot be used except to calibrate the former two.

Finally, we conclude that while aluminum- and polysilicon-based temperature sensors are not suitable to measure high microhotplate temperatures above 220 °C, they can be used to measure silicon substrate temperatures well below this temperature.

## Footnotes

1 Reference to commercial equipment is provided to fully describe the experimental procedure and does not constitute an endorsement by NIST nor a representation that the equipment is the best for the purpose.
